# Reduction of the Inflammatory Responses against Alginate-Poly-L-Lysine Microcapsules by Anti-Biofouling Surfaces of PEG-b-PLL Diblock Copolymers

**DOI:** 10.1371/journal.pone.0109837

**Published:** 2014-10-27

**Authors:** Milica Spasojevic, Genaro A. Paredes-Juarez, Joop Vorenkamp, Bart J. de Haan, Arend Jan Schouten, Paul de Vos

**Affiliations:** 1 Department of Polymer Chemistry, Zernike Institute for Advanced Materials, University of Groningen, Groningen, The Netherlands; 2 Departments of Pathology and Laboratory Medicine, section of Medical Biology, division of immunoendocrinology, University of Groningen, Groningen, The Netherlands; The Ohio State University, United States of America

## Abstract

Large-scale application of alginate-poly-L-lysine (alginate-PLL) capsules used for microencapsulation of living cells is hampered by varying degrees of success, caused by tissue responses against the capsules in the host. A major cause is proinflammatory PLL which is applied at the surface to provide semipermeable properties and immunoprotection. In this study, we investigated whether application of poly(ethylene glycol)-block-poly(L-lysine hydrochloride) diblock copolymers (PEG-b-PLL) can reduce the responses against PLL on alginate-matrices. The application of PEG-b-PLL was studied in two manners: (i) as a substitute for PLL or (ii) as an anti-biofouling layer on top of a proinflammatory, but immunoprotective, semipermeable alginate-PLL_100_ membrane. Transmission FTIR was applied to monitor the binding of PEG-b-PLL. When applied as a substitute for PLL, strong host responses in mice were observed. These responses were caused by insufficient binding of the PLL block of the diblock copolymers confirmed by FTIR. When PEG-b-PLL was applied as an anti-biofouling layer on top of PLL_100_ the responses in mice were severely reduced. Building an effective anti-biofouling layer required 50 hours as confirmed by FTIR, immunocytochemistry and XPS. Our study provides new insight in the binding requirements of polyamino acids necessary to provide an immunoprotective membrane. Furthermore, we present a relatively simple method to mask proinflammatory components on the surface of microcapsules to reduce host responses. Finally, but most importantly, our study illustrates the importance of combining physicochemical and biological methods to understand the complex interactions at the capsules' surface that determine the success or failure of microcapsules applicable for cell-encapsulation.

## Introduction

Microencapsulation of therapeutics cells is a promising approach for treatment of endocrine disorders such as anemia [1], dwarfism [2], hemophilia B [3], kidney [4] and liver [5] failure, pituitary [6] other central nervous system insufficiencies [7], and diabetes [8]. The semipermeable membrane allows for diffusion of nutrients and therapeutics, whereas the cells are protected from the immune system. This approach eliminates the necessity for immunosuppression and allows for xenografting. Xenografting may contribute to solving donor shortage.

Alginate-poly-L-lysine capsules have frequently been applied for microencapsulation of pancreatic islets [8]. Alginates are natural, unbranched polysaccharides composed of two monomer units, β-D-mannuronic acid (M) and its C-5 epimer, α-L-guluronic acid (G), connected by 1→4 linkages. They gel under physiological conditions without involvement of any toxic compounds such as harmful solvents. Many groups apply poly-L-lysine (PLL) to reduce the pore size and to provide immunoprotection [9], [10]. Normally unbound PLL is immunogenic [11]; however, to circumvent host responses against PLL, the microcapsules are ionically cross-linked with alginate to induce complexes of superhelical cores of alginate and PLL at the capsule's surface [12], [13]. But this process is not straightforward [14], [15]. Minor changes in the procedure can result in inadequate binding of proinflammatory PLL with strong immune reactions in the host as a consequence [13], [14], [16]–[18]. This was shown recently by our group in a comparison study of the *in vivo* behavior of a series of alginate-PLL capsules that differed only 10% in G-content. The alginate with higher G-content underwent changes *in vivo*, which resulted in the release of proinflammatory PLL followed by a strong tissue response [17].

Many different polycations have been proposed to substitute PLL, designed to provide immunoprotection on alginate matrixes for cell encapsulation [19]–[22]. Among them are chitosan [20], poly-L-ornithine [21], [23], poly-D-lysine [22] and diblock copolymers [24]. Often, however, new issues are introduced with these alternatives to PLL, leading again to severe inflammatory responses *in vivo* [19]. Partly, this is due to lack of knowledge about how the polyamino acids interact with alginate [14], [23], [25]–[27], but it is also due to the enormous lab-to-lab variations in successful formation of immunoprotective membranes [28], [29]. These issues led to our current proposal to design means for making capsule's surfaces more biocompatible, while still using PLL for providing immunoprotection because of its well known binding ability to alginate. Introducing diblock copolymers is, theoretically, such an approach, but has been difficult to achieve on the surface of cell-containing hydrophilic capsules. Many procedures to build membranes require harsh chemicals, eliminating them as options as only cell-friendly approaches may be applied to avoid loss of cells. The use of cell-friendly approaches is especially important when cell-sources from rare cadaveric donors are applied such as pancreatic islets for the treatment of diabetes [30]. Any loss of tissue is unacceptable in these types of applications. Here we studied the ability of PEG-b-PLL copolymers to reduce inflammatory responses. The copolymer can be applied as a complete substitute for PLL or as an additional layer on top of a preexisting proinflammatory PLL layer. The PEG-b-PLL copolymers can be bound to the surface of alginate without the application of chemicals that interfere with tissue viability. The PLL-block interacts ionically with the negatively charged alginate-core. The other block, polyethylene glycol (PEG), provides a biocompatible protecting layer on the surface of the capsules.

This study was designed to investigate the application of diblock copolymers in two manners. The first application was as a complete substitute for PLL, forming an immunoprotective membrane as previously suggested [24]. The other application was as an anti-biofouling layer on top of an immunoprotective PLL layer. We choose to use PLL_100_ to study the masking effects of the PEG-b-PLL copolymer. PLL_100_ provokes strong inflammatory responses due to incomplete binding to alginate as will be demonstrated in this study. The adsorption kinetics of the diblock copolymers on the alginate surface was studied by FTIR. The binding of diblock copolymers and surface properties in the absence and presence of the diblock copolymers were characterized by FTIR and XPS, respectively. Host responses were studied after implanted in the peritoneal cavity of balb/c mice.

## Materials and Methods

### Materials

Intermediate-G sodium alginate was obtained from ISP Alginates (UK). Poly-L-lysine hydrochloride (PLL_100_) (M_n_ = 16 kg/mol) and methoxy-poly(ethylene glycol)-block-poly(L-lysine hydrochloride) (PEG_x_-b-PLL_y_) (x = 454, M_n_ = 20 kg/mol; y = 50 or 100, M_n_ = 8 or 16 kg/mol; PDI = 1.2) were purchased from Alamanda Polymers (USA). Streptavidin fluorescein isothiocyanate (FITC) and Rabbit anti-PEG biotin were purchased from DakoCytomation (Denmark) and Bio-Connect B.V. (The Netherlands), respectively.

### Deposition of alginate films on silicon wafers

Prior to applying alginate coatings, double-side polished silicon wafers (Topsil Semiconductor Materials A/S, Frederikssund, Denmark 1000±15 µm thick) were cleaned by subsequent ultrasonication in dichloromethane, methanol, and acetone for 10 minutes. Residual organic contaminants were removed by UV-ozone treatment using an UV-ozone photoreactor PR-100 (Uvikon) for 60 minutes. Due to this treatment, the hydrophilicity of the exposed surface increases. Immediately after cleaning an alginate layer was applied on the surface.

Purified sodium alginate was dissolved in Krebs-Ringer-Hepes buffer (KRH, 220 mOsm) to give a 3.4 w/v % solution. The final alginate layer was obtained by dipping the recently cleaned and vertically aligned silicon wafers (1.5×1.0 cm) into the 3.4 w/v % alginate solution at a constant rate of 1 cm/min. The withdrawal rate was 10 cm/min. Silicon wafers coated with sodium alginate were placed into 100 m*M* CaCl_2_ buffer after which alginate was allowed to cross-link with calcium overnight. Before the alginate gels were exposed to PLL_100_ and copolymer solution, transmission FTIR spectra of the dry alginate layers were recorded.

The binding of copolymers to calcium alginate-PLL_100_ layers was studied as follows. After washing in KRH (containing 2.5 m*M* CaCl_2_) for 1 minute, one portion of alginate gel layers was incubated in PLL_100_ solution (in KRH containing 2.5 m*M* CaCl_2_, PLL concentration 6.25×10^−8^ mol/ml) for 10 minutes. Subsequently the layers were washed four times with KRH, dried under a filtered air stream and measured by FTIR. Alginate-PLL_100_ and the rest of alginate gel layers were incubated in copolymer solutions (in KRH containing 2.5 m*M* CaCl_2_, copolymer concentration 3.55×10^−8^ mol/ml). After certain time intervals, the wafers were removed from the copolymer solution, washed four times with KRH, dried under a filtered air stream and measured by FTIR. Subsequently the wafers were returned to the copolymer solution in order to continue the adsorption process and to determine the saturation point.

### Transmission Fourier transform infrared spectroscopy

The calcium alginate layers, as well as the layers after the pre-treatment with PLL_100_ and/or the adsorption of PEG-b-PLL copolymers, were studied by transmission FTIR. Measurements were performed under vacuum on a Bruker IFS 66 v/S spectrometer equipped with a DTGS detector and OPUS software package. A sample shuttle accessory was used for an interleaved sample and background scanning. A clean silicon wafer was used as a reference. All spectra are averages of 6×120 scans measured at a resolution of 4 cm^−1^.

The adsorption of PLL_100_ and the copolymer was followed by analyzing the increase in the surface area associated with asymmetric and symmetric C-H stretching vibrations (3000 to 2800 cm^−1^). In order to quantify the PLL- and copolymer-content on the calcium alginate, the surface area of the symmetric and asymmetric C-H stretching vibrations was determined. This value was reduced for the surface area corresponding to the C-H stretching vibrations of calcium alginate. Thus, the content of polymer attached to calcium alginate for each time point was obtained. These values were plotted as a function of time and the saturation point was determined as the starting point of the plateau.

### Microcapsules formation

Only intermediate-G alginates were used and were purified according to literature procedures [31]. Subsequently, capsules were produced based on a previously described procedure with some modifications [32], [33]. In some experiments cells were included. To this end, human insulin producing CM cells were cultured in RPMI (Gibco, Breda, The Netherlands) containing 60 kg/mL gentamicin and 10% heat-inactivated fetal calf serum (FCS) [34]. CM cells were always used between passage numbers 5 and 20. The cells were mixed at a concentration of 1×10^6^/ml with 3.4 w/v % sodium alginate solution. The cell containing or empty capsules were formed by converting the 3.4 w/v % sodium alginate solution into droplets using an air-driven generator [35]. The diameter of the droplets was controlled by a regulated airflow around the tip of needle. Alginate droplets were transformed to rigid alginate beads by gelling in a 100 m*M* CaCl_2_ solution for at least 10 minutes. The beads were washed with KRH (containing 2.5 m*M* CaCl_2_) for 1 minute. One portion of the beads was coated with the PEG-b-PLL copolymer for one hour and subsequently washed four times with KRH. Another portion of the beads was coated with PLL_100_ for 10 minutes (PLL_100_ solution in 310 mOsm KRH containing 2.5 m*M* CaCl_2_, PLL concentration 6.25×10^−8^ mol/mL), subsequently washed four times with KRH and in the last step the capsules were coated with the PEG-b-PLL copolymer for as long as required to obtain a saturated surface as monitored by FTIR. Finally, the capsules were washed 3 times with 310 mOsm KRH containing 2.5 m*M* CaCl_2_ and stored in this buffer. The diameters of capsules and beads were measured with a dissection microscope (Bausch and Lomb BVB-125, and 31–33–66) equipped with an ocular micrometer with an accuracy of 25 pm. The final diameter of the capsules was 600 µm.

### FITC labelling of microcapsules

Fluorescent labeling of microcapsules is a multi-step procedure. Primary antibody was added to a 10% solution of normal rabbit serum in phosphate buffered saline (PBS). The optimal primary antibody concentration was investigated and found to be when the antibody was diluted 500 times. To stain end-groups of PEG, 100 µl of this PBS solution was added to an eppendorf cup with approximately 20 capsules and left to shake for 1 hour at room temperature. The capsules were washed several times with PBS and subsequently incubated in PBS solution of streptavidin FITC (streptavidin FITC/PBS = 1/100) for 30 minutes in the dark. Finally, the capsules were washed several times with PBS, transferred onto a glass slide and studied at room temperature with a Leica TCS SP2 AOBS confocal microscope (50 w Hg lamp, HC PL APO CS 10×/0,30 dry, working distance 11 mm, 5(6)-FITC; FITC excitation wavelength 494 nm, FITC emission wavelength 518 nm). Confocal analyses were performed using the Imaris ×64 version 7.6.4 software.

### Testing cell viability

Viability of encapsulated cells was test using a LIVE/DEAD Cell Viability/Cytotoxicity assay Kit from InvitroGen, Life Technologies (New York, USA). Encapsulated cells were incubated for 30 min with Calcein AM (1 mM) and Ethidium Bromide (EB) (2 m*M*) at room temperature avoiding light. After incubation, the encapsulated cells were washed five times with KRH. Fluorescent confocal microscopy was measured at an emission wavelength of 517 nm (Calcein AM) and 617 nm (EB) using a Leica TCS SP2 AOBS confocal microscope (Wetzlar, Germany) equipped with an objective HC PL APO CS 10×/0,30, dry immersion, and working distance of 11 mm. Data was analyzed using Imaris ×64 version 7.6.4 software. The number of dead and live cells was quantified by counting at least 500 cells per batch. The fraction of dead cells was expressed as the percentage of the total number of counted cells.

### Diffusion characteristics

Permeability of capsules was studied using dextran-*f* samples of 10, 20, 40, 70, 110, or 150 kg/mol (TdB Consultancy AB, Sweden) as previously described [36]–[38]. For each dextran, approximately 50 capsules were placed on a microscope slide exposed to 200 µL of 0.1% dextran-*f* in Krebs Ringer Hepes, promptly covered with a glass coverslip and examined by fluorescence microscopy (Leica TCS SP2 AOBS confocal microscope). These permeability measurements were carried out in triplicate for each dextran-*f* MW.

### X-ray photoelectron spectroscopy (XPS)

In order to quantitatively study the atomic composition, samples of fresh capsules were washed three times with ultrapure water and gradually lyophilized. Samples of lyophilized capsules were fixed on a sample holder. The sample holder was inserted into the chamber of an X-ray photoelectron spectrometer (Surface Science Instruments, S-probe, Mountain View, CA). An aluminum anode was used for generation of X-rays (10 kV, 22 mA) at a spot size of 250×1000 µm. During the measurements, the pressure in the spectrometer was approximately 10^−7^ Pa. First, scans were collected over the binding energy range of 1–1100 eV at low resolution (150 eV pass energy). Next, we recorded at high resolution (50 eV pass energy) C_1s_, N_1s_, and O_1s_ peaks over a 20 eV binding energy range. The polymer content of the capsule's surface was expressed as a percentage of the total C, N, and O content of the membrane.

### Animal studies

Wild-type male Balb/c mice were purchased from Harlan (Harlan, Horst, The Netherlands). The animals were fed standard chow and water ad libitum. All animal experiments were performed after receiving approval of the institutional Animal Care Committee of the Groningen University. All animals received animal care in compliance with the Dutch law on Experimental Animal Care. The mice were sacrificed by cervical dislocation.

### Implantation and explanation of empty capsules

Capsules were injected into the peritoneal cavity with a 16 G cannula via a small incision (3 mm) in the linea alba. The abdomen was closed with a two-layer suture. The implanted volume was always 0.5 mL as assessed in a syringe with appropriate measure. The transplants contained at least 1000 capsules. The microcapsules were retrieved 1 month after implantation by peritoneal lavage. Peritoneal lavage was performed by infusing 2 mL KRH through a 3 mm midline incision into the peritoneal cavity and subsequent aspiration of the KRH containing the capsules. All surgical procedures were performed under isoflurane anesthesia.

### Histology

To assess the integrity of capsules before implantation, the samples of capsules were meticulously inspected for the presence of irregularities or defects in the capsule's membranes by using a dissection microscope.

To detect physical imperfections and to assess the composition and degree of overgrowth after implantation, samples of adherent capsules recovered by excision and samples of non-adherent capsules were fixed in pre-cooled 2% paraformaldehyde, buffered with 0.05 *M* phosphate in saline (pH 7.4), and processed for (hydroxyethyl)methacrylate (HEMA) embedding [39]. Sections were prepared at 2 µm, stained with Romanovsky-Giemsa stain and applied for detecting imperfections in the capsule's membrane, for quantifying the composition of the overgrowth and determining the number of capsules with and without overgrowth. Different cell-types in the overgrowth were assessed by identifying cells in the capsular overgrowth with the morphological characteristics of monocytes/macrophages, lymphocytes, granulocytes, fibroblasts, basophiles, erythrocytes, and multinucleated giant cells. To confirm the adequacy of this approach, portions of adherent and non-adherent capsules were frozen in precooled isopropane as described in a previous study [17], sectioned at 5 µm, and processed for immunohistochemical staining and quantification of the different cell types as previously described [40]. The used monoclonal antibodies were: ED1 and ED2 against monocytes and macrophages [41], HIS-40 against IgM bearing B-lymphocytes [42], and R73 against CD3^+^ bearing T-lymphocytes [43], In control sections we used PBS instead of the first stage monoclonal antibody. Quantification of these cells types after immunocytochemistry was compared with the assessments on the basis of morphological markers and always gave similar results.

The degree of capsular overgrowth was quantified by expressing the number of recovered capsules with overgrowth as the percentage of the total number of recovered capsules for each individual animal.

### Statistical analysis

Values are expressed as mean ± standard error of the mean (SEM). Normal distribution of the data was confirmed using the Kolmogorov-Smirnov test. As no normal distribution could be demonstrated, we applied the nonparametric Mann Whitney-U test. P-values<0.05 were considered to be statistically significant. The n-values for the animal experiments were based on a mandatory power analysis. The values were 4 mice per experimental group, based on a type I error of 5% and a type II error of 10%.

## Results

### The host responses against alginate-capsules where the PLL layer was completely substituted by PEG_454_-b-PLL_100_ to provide immunoprotection

**Figure 1:**
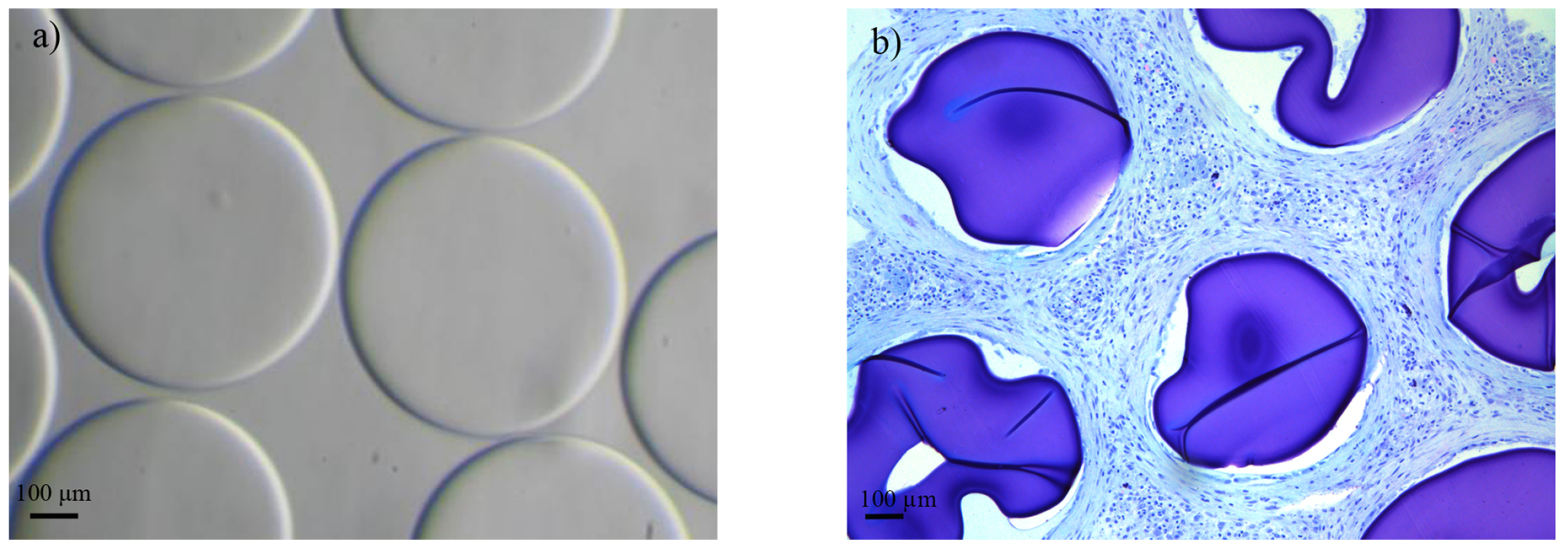
Alginate-PEG_454_-b-PLL_100_ capsules a) before implantation and b) at one month after implantation. GMA-embedded histological sections, Romanovsky-Giemsa staining, original magnification ×10.

Based on previous findings [24], we chose the long PEG_454_ for the *in vivo* application because these long chains cannot easily penetrate into the alginate matrix and will stay at the surface. The positively charged PLL blocks are relatively small and will readily penetrate the alginate matrix where the ammonium groups of PLL will ionically interact with the carboxyl groups of alginate. To this end, the two PEG-b-PLL diblock copolymers were allowed to cross-link for one hour. This time period has found to be sufficient to create capsules with a permeability that does not allow entry of molecules larger than 120 kg/mol, which is considered to be an immunoprotective threshold [24], [31], [44]. Before implantation all capsules were meticulously microscopically inspected. Only perfect capsules with no tails or other imperfections associated with host responses were selected for implantation [45]–[47] (Figure 1a).

**Figure 2:**
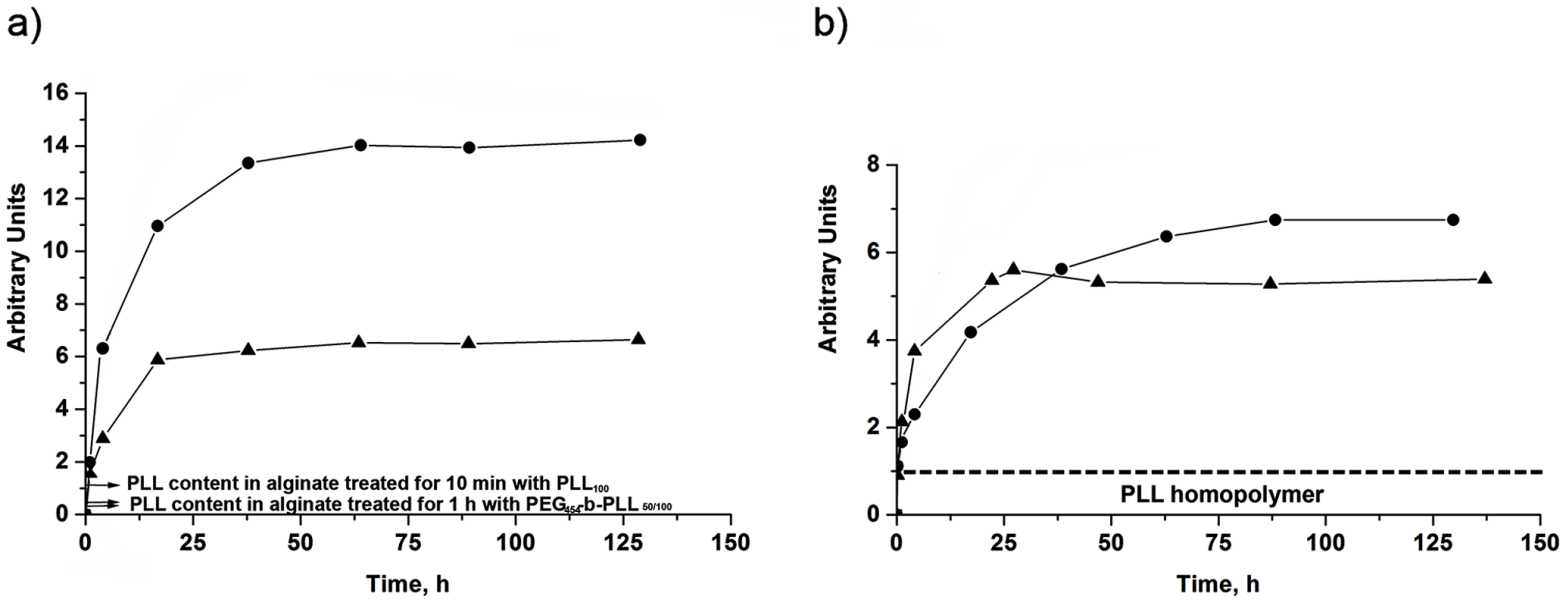
Kinetics of adsorption of the PEG_454_-b-PLL_50_ (•) and PEG_454_-b-PLL_100_ (▴) diblock copolymer on a) the alginate gel and b) the alginate gel pretreated for 10 minutes with PLL_100_.

The capsules were implanted in the peritoneal cavity of balb/c mice and retrieved after one month. Macroscopically, the capsules with either an immunoprotective PEG_454_-b-PLL_50_ or PEG_454_-b-PLL_100_ were found in one large clump around the place of implantation. Examination by histology revealed that the capsules were caught in thick layers of fibroblast and were adherent to each other. This may be a sign of an unstable membrane in which positively charged molecules instantly attract inflammatory cells leading to heavy fibroblast overgrowth (Figure 1b). A series of infrared studies revealed that the relatively short period of incubation (*i.e.* 1 hour), which provides a permeability of 100–120 kg/mol [24] with PEG_454_-b-PLL_y_ (y = 50 or 100), was too short to allow the formation of a stable membrane (see Figure 2a).

**Figure 3:**
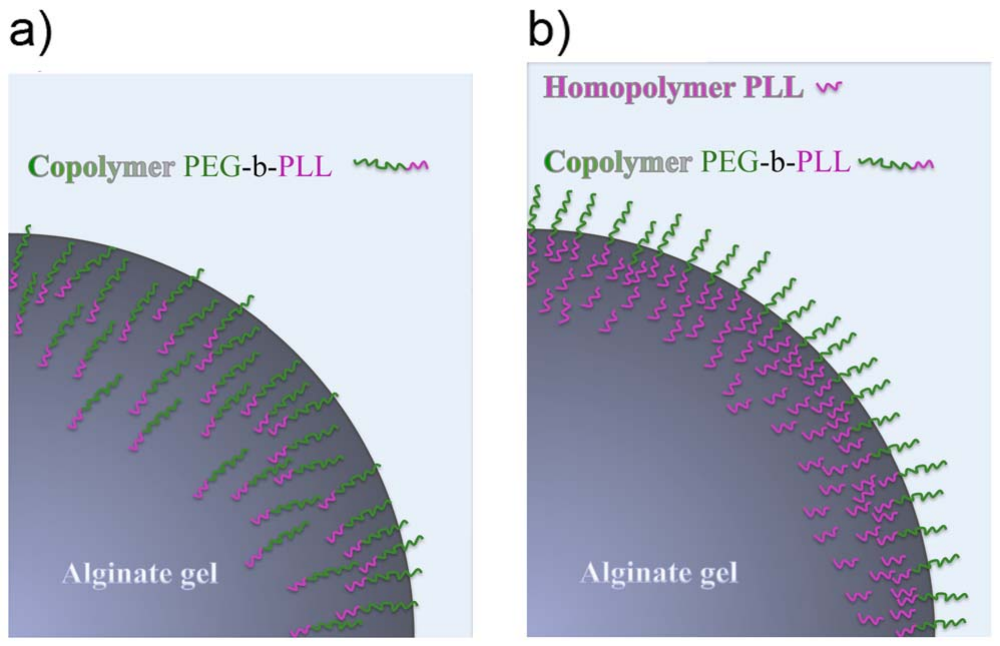
Illustration of a) alginate-PEG-b-PLL capsules (without PLL_100_ pretreatment) and b) alginate-PLL-PEG-b-PLL capsules (with PLL_100_ pretreatment).

The fact that both PEG_454_-b-PLL_y_ (y = 50 or 100) cannot adequately substitute PLL in providing an immunoprotective membrane does not imply that they cannot be used for other purposes. The copolymers can be used for the formation of a masking anti-biofouling layer on top of PLL. PEG-b-PLL copolymers have been characterized as polymer with a low immunogenic capacity as they do elicit minor immune activation of nuclear factor NF-κB in THP-1 monocytes [24]. A prerequisite as outlined above, is that the diblock copolymer chains should be adequately bound to the matrix. For these reasons, the next step in our study was to apply PEG-b-PLL copolymers on top of a preexisting immunoprotective layer of proinflammatory PLL. Prior to the copolymer treatment, PLL_100_ was applied to reduce the permeability of the alginate beads. This was done according to the principle illustrated in Figure 3. PLL_100_ efficiently reduces permeability, but PLL_100_ does provoke strong host responses as shown below. In order to determine the time period required to build an effective copolymer layer on top of the alginate-PLL_100_ membrane, we applied FTIR. To this end, one to 1.5 µm thick alginate layers deposited on silicon wafers were incubated in a PLL_100_ solution for 10 minutes, measured by FTIR and subsequently exposed to the copolymer solution and measured again. The kinetics of the adsorption was followed through the increase of the bands that correspond to symmetric and asymmetric C-H stretching vibrations in the FTIR spectrum. Since methyl, methylene, and methine groups do not participate in hydrogen bonding, the position of the bands corresponding to these groups is virtually not influenced by the chemical environment of the measured substance [48]. Therefore, this region was considered as the most reliable to study the quantity of the adsorbed PLL and/or copolymers. The surface area of the C-H bands was determined, reduced for the value which corresponds to C-H vibrations of the alginate gel and plotted as a function of time (see Figure 2).

After the pretreatment of the calcium-alginate layers with PLL_100_, FTIR analysis showed that diblock copolymer chains could still interact and bind to the alginate gels as illustrated in Figure 3b. Binding of copolymers to the alginate-PLL_100_ layer started immediately, continued asymptotically and reached a maximum value after approximately 25 hours for PEG_454_-b-PLL_100_ and 50 hours for PEG_454_-b-PLL_50_ (Figure 2b). Consequently, these time periods were taken as the minimum to achieve a high concentration of copolymers on the capsules' surface and to form an anti-biofouling layer on top of the alginate-PLL_100_ layer.

**Figure 4:**
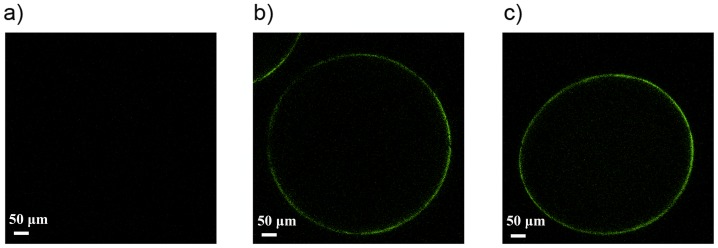
Confocal microscopy images after staining of the PEG blocks. **a) Alginate-PLL_100_ capsules, b) alginate-PLL_100_-PEG_454_-b-PLL_50_ capsules and c) alginate-PLL_100_-PEG_454_-b-PLL_100_ microcapsules.** Original magnification 10×.

In the present study we compared the capsules coated with the diblock copolymers for one hour with capsules coated with PLL_100_ (10 minutes) and with PEG_454_-b-PLL_50_ for 50 hours. The reason is that we took the saturation time periods and therefore made this comparison. The alginate-PLL_100_-PEG_454_-b-PLL_y_ (y = 50 or 100) capsules were prepared by incubating alginate beads in the PLL_100_ solution for 10 min and subsequently in the copolymer solution for approximately 50 hours. To confirm binding of copolymers to PLL_100_-precoated alginate capsules, the staining of the PEG blocks at the surface with antibodies directed against the end group of these blocks (methoxy group) was performed. PLL_100_ capsules were used as negative control. The presence of green fluorescence on the alginate-PLL_100_-PEG_454_-b-PLL_y_ (y = 50 or 100) microcapsules demonstrated successful adsorption of diblock copolymers on the surface (Figure 4).

**Figure 5:**
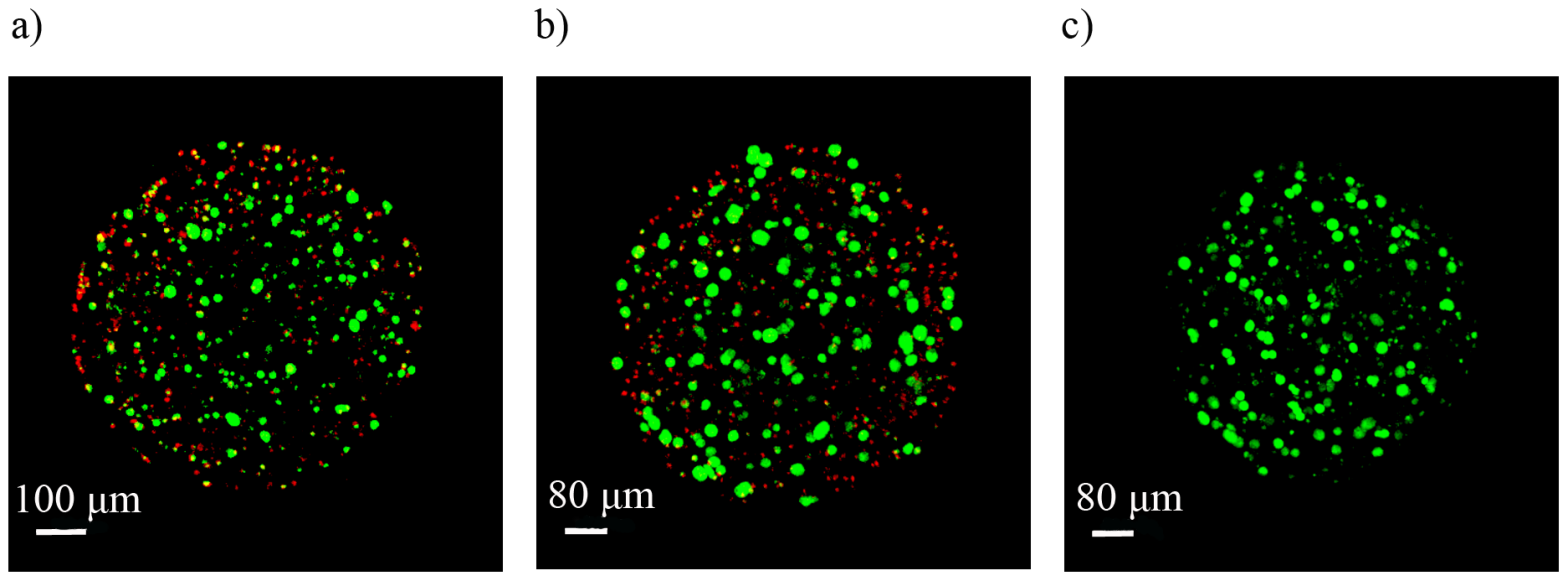
Viability of the insulin producing CM-cells encapsulated in a) alginate-PLL_100_ capsules, b) alginate-PLL_100_-PEG_454_-b-PLL_50_ capsules and c) alginate-PLL_100_-PEG_454_-b-PLL_100_ microcapsules after 5 days of culturing. The remnants and dead cells were still visible in the periphery of the capsules.

In order to determine whether long incubation times of 50 hours can influence the viability of cells, the insulin producing CM-cells were encapsulated according to this new procedure. CM-cells encapsulated in conventional control alginate-PLL_100_ capsules, that were exposed for only ten minutes to PLL, served as control. The cell-containing capsules were subjected to live-dead staining for studying by confocal microscopy after the encapsulation procedure as well as after culturing for 5 days.. Figure 5 shows the results. The number of dead cells in the capsules was always below 20% and was not different between the freshly encapsulated cells and cells in capsules incubated for 5 days (Table 1). As shown in the enclosed Movie S1 after 5 days of culturing only the remnants of dead cells were still visible. The remnants and dead cells were always in the periphery of the capsules and were observed in all capsule types suggesting that direct interaction with PLL rather than the incubation times is responsible for death of these cells. The same results (data not shown) were obtained for T84 cells which usually are very sensitive for long times of serum deprivation.

**Table 1:** Percentage of dead CM-cells encapsulated in a) alginate-PLL_100_ capsules (10 minutes incubation), b) alginate-PLL_100_-PEG_454_-b-PLL_50_ capsules (50 hours incubation) and c) alginate-PLL_100_-PEG_454_-b-PLL_100_ microcapsules (50 hours incubation) immediately after encapsulation and after 5 days of culturing (n = 4).

Samples of capsules	Dead CM-cells	
	Direct after encapsulation	Five days after encapsulation
Alginate-PLL100	15.75±1.80	14±3.39
Alginate-PLL_100_-PEG_454_-b-PLL_50_	17.25±3.47	8.5±2.40
Alginate-PLL_100_-PEG_454_-b-PLL_100_	17.75±3.79	12±2.12

**Figure 6:**
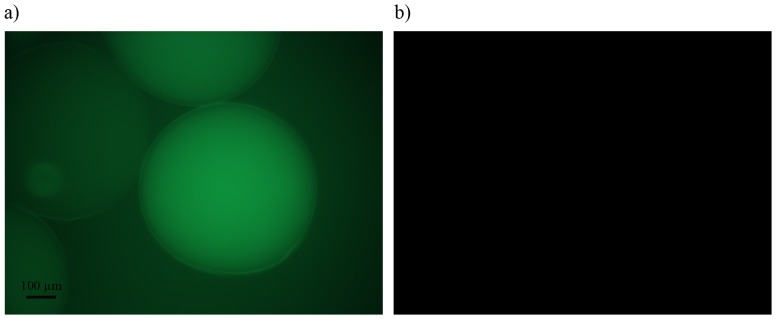
Confocal microscopy images of alginate-PLL-PEG-b-PLL microcapsules after the addition of a) dextran of 110 kg/mol and b) dextran of 150 kg/mol.

The coating procedure had no influence on the permeability of the capsules. The alginate-PLL_100_ capsules, as well as the 25 hours PEG_454_-b-PLL_100_ and the 50 hours for PEG_454_-b-PLL_50_ capsules were tested for permeability with fluorescent dextran with molecular weights of 10, 20, 40, 70, 110, and 150 kg/mol. All three capsule's types were still allowing entry of dextran with a molecular weight of 110 kg/mol but were impermeable for dextran with a Mw of 150 kg/mol (Table 2 and Figure 6). Uncoated, calcium alginate beads were permeable for all samples of dextran. This illustrated that the initial PLL_100_ incubation is the diffusion-limiting step.

**Table 2:** Permeability of the alginate-PLL_100_, alginate-PLL_100_-PEG_454_-b-PLL_50_ and alginate-PLL_100_-PEG_454_-b-PLL_100_ capsules determined using dextran-*f* samples.

Dextran Samples, Molecular weight of dextran, kg/mol	Type of the alginate capsules (A)		
	A-PLL_100_	A-PLL_100_-PEG_454_-b-PLL_50_	A-PLL_100_-PEG_454_-b-PLL_100_
10	+	+	+
20	+	+	+
40	+	+	+
70	+	+	+
110	+	+	+
150	−	−	−

### X-ray photoelectron spectroscopy confirms presence of diblock copolymers at the surface

X-ray photoelectron spectroscopy (XPS) is a surface-sensitive quantitative technique for studying elemental composition, chemical, and electronic state of the elements in the material. This technique provides information for the top 2 to 10 nm of any analyzed material. XPS has been extensively used to study the composition of the capsule's surface [15], [17], [26], [49]. To investigate the elemental composition, capsules were analyzed by XPS [17].

The surface elemental composition of the alginate-PLL_100_ and alginate-PLL_100_-PEG_454_-b-PLL_y_ (y = 50 or 100) capsules is presented in Table 3. The ratio of carbon to nitrogen (C/N) for the surface of the PLL-microcapsules was 8.14, whereas the theoretical C/N ratio for PLL is 3. This indicates that 2–10 nm surface layer is composed of both alginate and PLL as shown in our previous studies [17], [49]. The C/N ratio for the surface of the alginate-PLL_100_-PEG_454_-b-PLL_y_ (y = 50 or 100) capsules is similar to the theoretical C/N ratio of the corresponding copolymers. Therefore, the XPS analysis confirmed that the surface of these capsules is mainly composed of the diblock copolymers.

**Table 3:** Elemental surface compositions of alginate-PLL_100_ and alginate-PLL_100_-PEG_454_-b-PLL_y_ (y = 50 or 100) microcapsules and theoretical atom % of PLL_100_ homopolymer and PEG_454_-b-PLL_y_ (y = 50 or 100) diblock copolymers.

Capsules, alginate-	C, %	O, %	N, %	Ca, %	Others (Including Na and Cl), %	C/N ratio
PLL_100_	58.42	26.97	7.18	1.38	6.05	8.14
PLL-PEG_454_-b-PLL_50_	66.38	27.37	6.25	0	0	10.62
PLL-PEG_454_-b-PLL_100_	65.70	25.26	9.04	0	0	7.27
**Theoretical atom % of**						
PLL	66.67	11.11	22.22	0	0	3.00
PEG	66.67	33.33	0	0	0	-
PEG_454_-b-PLL_50_	66.67	27.81	5.52	0	0	12.08
PEG_454_-b-PLL_100_	66.67	24.49	8.84	0	0	7.54

### Host response against alginate-PLL_100_ and alginate-PLL_100_-PEG_454_-b-PLL_50_ capsules

The last step in our study was to investigate whether the copolymer layer, formed after up to 50 hours of cross-linking with alginate-PLL_100_ was functional *in vivo*. We only applied the PEG_454_-b-PLL_50_ in the *in vivo* study. Alginate-PLL_100_ capsules (*i.e.* controls) and the alginate-PLL_100_-PEG_454_-b-PLL_50_ capsules were implanted in the peritoneal cavity of balb/c mice. Before implantation, the grafts (n = 4) were meticulously inspected to ensure that they had a similar mechanical stability and had no broken or imperfect capsules.

**Figure 7:**
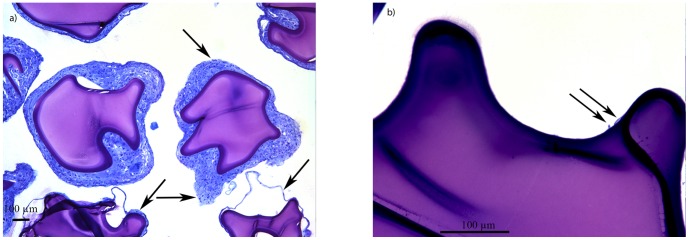
Explanted a) alginate-PLL_100_, (original magnification 10×). Note the macrophages and fibroblasts. b) Alginate-PLL_100_-PEG_454_-b-PLL_50_ microcapsules (original magnification 40×). Only a portion of capsules had inflammatory cells at the surface. Note that the affected capsules in most cases had adherence of a few or sometimes clumps of cells instead of complete coverage as in a). This suggests that local imperfections at the capsule's surface may be responsible for cell adhesion. All capsules were retrieved one month after implantation in the peritoneal cavity of balb/c mice GMA-embedded histological sections, Romanovsky-Giemsa staining.

The alginate-PLL_100_ capsules without an anti-biofouling layer provoked a very strong inflammatory response as expected. All capsules were found to adhere to the surface of the abdominal organs, which caused a low retrieval rate of the capsules (Figure 7a). In two animals the capsules were found as clumps on top of the liver and were completely caught in thick layers of fibroconnective tissue. Histologically high numbers of macrophages and fibroblasts were found. We also found multinucleated giant cells but no T-cells or B-cells. The few alginate-PLL_100_ capsules that escaped from the host response where mostly caught in the fibrotic clumps.

This was different when the anti-biofouling layer of PEG_454_-b-PLL_50_ was applied (Table 4). Upon retrieval, 80–100% of the capsule grafts were recovered from the peritoneal cavity, whereas only 2.5±5% of the alginate-PLL_100_ capsules were recovered (P<0.01). The alginate-PLL_100_-PEG_454_-b-PLL_50_ capsules were mostly free-floating and did not adhere to the abdominal organs. The capsules were found in between the intestines and clumping was rarely observed [50]. The percentage of capsules with cellular overgrowth with alginate-PLL_100_-PEG_454_-b-PLL_50_ capsules was 36.25±27.87% whereas with alginate-PLL_100_ capsules it was 97.25±5.5% (P<0.01) at one month after implantation. The capsules' surface was only rarely covered completely with the cellular overgrowth. Mostly, just a few cells were adhered which is usually interpreted as a local imperfection on the capsules' surface. The overgrowth was mainly composed of macrophages and a few fibroblasts (Figure 7b). We found no T-cells or other cells of the adaptive immune system on the capsules or on surrounding tissues that were taken for biopsy.

**Table 4:** Recovery rates and percentage of alginate-PLL_100_ and alginate-PLL_100_–PEG_454_-b-PLL_50_ capsules with overgrowth, 1 month after implantation in the peritoneal cavity of balb/c mice.

Type of capsules	n	Recovery, %	Overgrowth, %
Alginate-PLL_100_	4	2.5±5	97.25±5.5
Alginate-PLL_100_-PEG_454_-b-PLL_50_	4	95±10	36.25±27.87

## Discussion

A combined physicochemical and biological approach is still rarely implied in the encapsulation field [28]. The observation that by using diblock copolymers as substitutes for PLL strong inflammatory responses were induced while the diblock copolymers applied on the top of the alginate-PLL_100_ surface reduced inflammatory responses, illustrates the necessity of a multidisciplinary approach in understanding the chemical background of host responses against microcapsules. Our work demonstrates that some polymers such as PEG_454_-b-PLL_50_ or PEG_454_-b-PLL_100_ are not applicable for creating immunoprotective membranes. The relatively short incubation times required to create a membrane impermeable for molecules above 100–120 kg/mol are not sufficient to provide stable membranes. The same may hold true for many other polymers suggested to substitute PLL [20]–[23].

In this study, only intermediate-G alginates were applied, as only this type of alginate contains sufficient G-M blocks to bind PLL [17], [32]. The diblock copolymer had no effect on the cells in the matrix as demonstrated with insulin producing CM-cells. Moreover, the PEG-b-PLL copolymer has been characterized as a unique polymer with a low immunogenic capacity [24], and PEG is known to provide an anti-biofouling layer in cell microencapsulation [51]–[56]. Therefore, we did not immediately abandon its application. Instead we studied whether the copolymer can form an anti-biofouling layer on top of the capsules' surface, which should reduce host responses against capsule's components. However, before studying the application of the copolymers as anti-biofouling layer on top of PLL_100_, we first did a chemical analysis of the capsules' surface and determined the requirements for the optimal binding. Transmission FTIR study was applied to determine the time-period required for optimal binding and saturation. Elemental analysis of the capsules' surface in combination with immunocytochemistry demonstrated the efficiency of the bound copolymers to mask proinflammatory PLL. We found that 50 hours of incubation were required to form an efficacious layer on top of the PLL_100_. Such long incubation time-periods may not be applicable for all cell types, but up to now all cells we applied did survive and functioned when cultured for prolonged periods in Krebs-Ringer-Hepes (KRH). KRH is a balanced salt solution that was especially developed for encapsulation of cells [33]. It is serum free but allows for survival of cells for prolonged periods of time.

The PEG_454_-b-PLL_50_ binding severely reduced the responses in mice against the alginate-PLL_100_ surfaces. The vast majority of the alginate-PLL_100_-PEG_454_-b-PLL_50_ capsules were free of any cell adhesion and free-floating in the peritoneal cavity, whereas nearly all alginate-PLL_100_ capsules without the copolymer were completely overgrown with macrophages and fibroblasts. Notably, however, some attachment of inflammatory cells was still observed on a portion of the alginate-PLL_100_-PEG_454_-b-PLL_50_ capsules. This adhesion of cells was different from what we have previously observed [15], [57]–[60]. Complete coverage of capsule with inflammatory cells and fibroblasts, which is indicative for a foreign body response to the capsules, was rarely observed. In most cases, adhesion of groups of macrophages to specific parts of the capsule's surface was seen, suggesting that local imperfections were responsible for immune activation [45], [61]. We believe that spatial differences in coating efficacy can be the cause of this type of cell adhesion implying that the system may still be improved in spite of the step-wise chemical approach. For sake of clarity, we counted all the capsules with overgrowth irrespective of the degree of overgrowth. Sometimes just one or two cells were found on the capsules with the PEG_454_-b-PLL_50_ copolymer (Figure 7b). We believe that these cells will not have an influence on the functional survival of the cells in the capsules [45], [61]. The data should therefore be carefully interpreted. The overgrowth is not necessarily having more consequences for cell survival than what was observed in previous studies were around 10% of the capsules were affected but infiltrated with large numbers of inflammatory cells instead of the few cells we found on the affected capsules in this study [44], [45], [62].

Creating an immunoprotective membrane with PLL without causing an inflammatory response has been shown to be a pitfall in many laboratories [27], [63]. Variations in creating an efficacious PLL-membrane that provides immunoprotection without host-responses are one of the major factors responsible for the reported lab-to-lab variations with microcapsules [11], [28], [44], [49], [60], [64]. The role of PLL in host responses has also been demonstrated in studies that show that calcium alginate normally does not provoke a response, but as soon as a polyamino acid is applied, strong inflammatory responses arise [63]. Adequate binding of PLL on the alginate matrix, which should result in formation of superhelical cores of alginate around PLL, depends on several crucial factors [14], [29], [65]. It is well recognized that alginate should contain sufficient G-M residues to bind all proinflammatory PLL [17], [66]. A seemingly minor difference in G-M content can lead to leakage of PLL *in vivo* with foreign body responses as a consequence [17]. Another factor that is not often taken into consideration is the porosity of the alginate-gel in relation to the size of PLL chain. In our lab the 3.4% intermediate-G alginate gels are commonly used to create an immunoprotective membrane in combination with PLL of 22 to 24 kg/mol [15], [17], [49]. This relatively large molecule will only bind to sodium-alginate residues at the top 2–4 µm surface of the capsules [15], [19], [28]. Lower alginate concentrations or smaller PLL molecules can cause incomplete binding of PLL to the alginate core followed by leakage or exposure of unbound PLL at the capsule's surface *in vivo* with eventually host-responses as a consequence [11], [13], [67]. As shown here, anti-biofouling layers of the PEG_454_-b-PLL_50_ copolymer may contribute to making PLL binding a less delicate process. Building an efficacious antifouling layer requires however a long incubation period of 50 hours, but it is rather simple as it involves only an incubation step. The binding efficacy can easily be followed through the increase of the bands that correspond to symmetric and asymmetric C-H stretching vibrations in the FTIR spectrum. The simple incubation step requires much less skills and technologies than adequate binding of PLL which depends not only on incubation with PLL but also on exchange of series of ions [14]. The application of this anti-biofouling layer may reduce in the enormous lab-to-lab variations that are considered to be a major threat for progress in the field [17], [29], [68].

Our study should not be interpreted as a suggestion that PLL binding is the only factor in host-responses against alginate-based microcapsules. Other important issues are the degree of purity of the alginates [16], [26], [28], [65] and the type of alginates [18], [32], [49], [62], [64]. Crude alginates contain not only polyphenols but also pathogen associated molecular patterns that are potent stimulators of the immune system [69], [70]. Nowadays, only ultrapure alginates are applied and intermediate-G alginates are preferred over high-G alginates despite a better mechanical stability of the high-G alginate gels [32], [71]–[74]. In this study, only pure alginates with no immunostimulatory capacity were applied [24]. Our data showed that in spite of the extreme purity of alginates, inflammatory responses against capsules still occur due to presence of positively charged polyamino acids at the surface of capsules that are not in the required confirmation [12], [13].

## Conclusions

PEG-b-PLL diblock copolymers may contribute to reduction of host responses against alginate-PLL_100_ capsules by masking proinflammatory PLL_100_ residues. As such, PEG-b-PLL diblock copolymers are effective anti-biofouling molecules. Also, it was demonstrated that PEG-b-PLL diblock copolymers are not suitable as complete substitute for PLL because they provide membranes with the corresponding permeability but are unstable *in vivo*. Our study further illustrates the necessity of combining physicochemical and biological means to understand the complex interactions at the surface of microcapsules and the associated biological responses.

## Supporting Information

Movie S1 **Viability of the insulin producing CM-cells encapsulated in alginate-PLL_100_ capsules after 5 days of culturing.** After 5 days of culturing only the remnants of dead cells were still visible. The remnants and dead cells were always in the periphery of the capsules suggesting that direct interaction with PLL rather than the incubation times is responsible for death of these cells.
(AVI)

## References

[1] Koo J, Chang TM (1993) Secretion of erythropoietin from microencapsulated rat kidney cells: preliminary results. Int J Artif Organs 16: 557–560. 8370612

[2] Chang PL, Shen N, Westcott AJ (1993) Delivery of recombinant gene products with microencapsulated cells in vivo. Hum Gene Ther 4: 433–440. 8399490 10.1089/hum.1993.4.4-433

[3] Liu HW, Ofosu FA, Chang PL (1993) Expression of Human Factor IX by Microencapsulated Recombinant Fibroblasts. Hum Gene Ther 4: 291–301. 8338876 10.1089/hum.1993.4.3-291

[4] Cieslinski DA, David Humes H (1994) Tissue engineering of a bioartificial kidney. Biotechnol Bioeng 43: 678–681. 18615768 10.1002/bit.260430718

[5] Uludag H, Sefton MV (1993) Metabolic activity and proliferation of CHO cells in hydroxyethyl methacrylate-methyl methacrylate (HEMA-MMA) microcapsules. Cell Transplant 2: 175–182. 8143082 10.1177/096368979300200210

[6] Colton CK (1995) Implantable biohybrid artificial organs. Cell Transplant 4: 415–436. 7582573 10.1177/096368979500400413

[7] Aebischer P, Goddard M, Signore AP, Timpson RL (1994) Functional recovery in hemiparkinsonian primates transplanted with polymer-encapsulated PC12 cells. Exp Neurol 126: 151–158. 7925816 10.1006/exnr.1994.1053

[8] Lim F, Sun AM (1980) Microencapsulated islets as bioartificial endocrine pancreas. Science 210: 908–910. 6776628 10.1126/science.6776628

[9] Leblond FA, Tessier J, Hallé J-P (1996) Quantitative method for the evaluation of biomicrocapsule resistance to mechanical stress. Biomaterials 17: 2097–2102. 8902243 10.1016/0142-9612(96)00027-0

[10] Robitaille R, Leblond FA, Bourgeois Y, Henley N, Loignon M, et al (2000) Studies on small (<350 µm) alginate-poly-L-lysine microcapsules. V. Determination of carbohydrate and protein permeation through microcapsules by reverse-size exclusion chromatography. J Biomed Mater Res 50: 420–427. 10737885 10.1002/(sici)1097-4636(20000605)50:3<420::aid-jbm17>3.0.co;2-s

[11] Strand BL, Ryan TL, In't Veld P, Kulseng B, Rokstad AM, et al (2001) Poly-L-Lysine induces fibrosis on alginate microcapsules via the induction of cytokines. Cell Transplant 10: 263–275. 11437072

[12] Uludag H, De Vos P, Tresco PA (2000) Technology of mammalian cell encapsulation. Adv Drug Delivery Rev 42: 29–64. 10.1016/s0169-409x(00)00053-3 10942814

[13] Vandenbossche GMR, Bracke ME, Cuvelier CA, Bortier HE, Mareel MM, et al (1993) Host Reaction against Empty Alginate-polylysine Microcapsules. Influence of Preparation Procedure. J Pharm Pharmacol 45: 115–120. 8095525 10.1111/j.2042-7158.1993.tb03694.x

[14] van Hoogmoed CG, Busscher HJ, de Vos P (2003) Fourier transform infrared spectroscopy studies of alginate-PLL capsules with varying compositions. J Biomed Mater Res A 67: 172–178. 14517874 10.1002/jbm.a.10086

[15] de Vos P, van Hoogmoed CG, van Zanten J, Netter S, Strubbe JH, et al (2003) Long-term biocompatibility, chemistry, and function of microencapsulated pancreatic islets. Biomaterials 24: 305–312. 12419632 10.1016/s0142-9612(02)00319-8

[16] de Haan BJ, Rossi A, Faas MM, Smelt MJ, Sonvico F, et al (2011) Structural surface changes and inflammatory responses against alginate-based microcapsules after exposure to human peritoneal fluid. J Biomed Mater Res A 98A: 394–403. 10.1002/jbm.a.33123 21630432

[17] de Vos P, Spasojevic M, de Haan BJ, Faas MM (2012) The association between in vivo physicochemical changes and inflammatory responses against alginate based microcapsules. Biomaterials 33: 5552–5559. 22560199 10.1016/j.biomaterials.2012.04.039

[18] de Vos P, de Haan BJ, Kamps JA, Faas MM, Kitano T (2007) Zeta-potentials of alginate-PLL capsules: a predictive measure for biocompatibility? J Biomed Mater Res A 80: 813–819. 17058213 10.1002/jbm.a.30979

[19] Ponce S, Orive G, Hernández R, Gascón AR, Pedraz JL, et al (2006) Chemistry and the biological response against immunoisolating alginate–polycation capsules of different composition. Biomaterials 27: 4831–4839. 16766026 10.1016/j.biomaterials.2006.05.014

[20] Orive G, Bartkowiak A, Lisiecki S, De Castro M, Hernández RM, et al (2005) Biocompatible oligochitosans as cationic modifiers of alginate/Ca microcapsules. J Biomed Mater Res B Appl Biomater 74: 429–439. 15909303 10.1002/jbm.b.30146

[21] Basta G, Sarchielli P, Luca G, Racanicchi L, Nastruzzi C, et al (2004) Optimized parameters for microencapsulation of pancreatic islet cells: an in vitro study clueing on islet graft immunoprotection in type 1 diabetes mellitus. Transpl Immunol 13: 289–296. 15589742 10.1016/j.trim.2004.10.003

[22] Bystrický S, Malovíková A, Sticzay T (1991) Interaction of acidic polysaccharides with polylysine enantiomers. Conformation probe in solution. Carbohydr Polym 15: 299–308.

[23] Tam SK, Bilodeau S, Dusseault J, Langlois G, Hallé JP, et al (2011) Biocompatibility and physicochemical characteristics of alginate–polycation microcapsules. Acta Biomater 7: 1683–1692. 21145438 10.1016/j.actbio.2010.12.006

[24] Spasojevic M, Bhujbal S, Paredes G, de Haan BJ, Schouten AJ, et al (2013) Considerations in binding diblock copolymers on hydrophilic alginate beads for providing an immunoprotective membrane. J Biomed Mater Res A 102: 1887–1896. 23853069 10.1002/jbm.a.34863 PMC4232034

[25] Rokstad AMA, Lacík I, de Vos P, Strand BL (2013) Advances in biocompatibility and physico-chemical characterization of microspheres for cell encapsulation. Adv Drug Deliv Rev 67–68: 111–130. 10.1016/j.addr.2013.07.010 23876549

[26] Tam SK, Dusseault J, Bilodeau S, Langlois G, Hallé J-P, et al (2011) Factors influencing alginate gel biocompatibility. J Biomed Mater Res A 98A: 40–52. 10.1002/jbm.a.33047 21523903

[27] Rokstad AM, Brekke O-L, Steinkjer B, Ryan L, Kolláriková G, et al (2013) The induction of cytokines by polycation containing microspheres by a complement dependent mechanism. Biomaterials 34: 621–630. 23103159 10.1016/j.biomaterials.2012.10.012

[28] de Vos P, Buèko M, Gemeiner P, Navrátil M, Švitel J, et al (2009) Multiscale requirements for bioencapsulation in medicine and biotechnology. Biomaterials 30: 2559–2570. 19201460 10.1016/j.biomaterials.2009.01.014

[29] Orive G, Emerich D, de Vos P (2014) Encapsulate this: the do's and don'ts. Nat Med 20. 10.1038/nm.3486 24603789

[30] Bruns H, Schultze D, Schemmer P (2013) Alternatives to islet transplantation: future cell sources of beta-like cells. Clin Transplant 27: 30–33. 23909499 10.1111/ctr.12153

[31] De Vos P, De Haan BJ, Wolters GH, Strubbe JH, Van Schilfgaarde R (1997) Improved biocompatibility but limited graft survival after purification of alginate for microencapsulation of pancreatic islets. Diabetologia 40: 262–270. 9084963 10.1007/s001250050673

[32] De Vos P, De Haan B, Van Schilfgaarde R (1997) Effect of the alginate composition on the biocompatibility of alginate-polylysine microcapsules. Biomaterials 18: 273–278. 9031730 10.1016/s0142-9612(96)00135-4

[33] de Haan BJ, Faas MM, de Vos P (2003) Factors influencing insulin secretion from encapsulated islets. Cell Transplant 12: 617–625. 14579930 10.3727/000000003108747226

[34] Smelt MJ, Faas MM, de Haan BJ, Draijer C, Hugenholtz GC, et al (2012) Susceptibility of human pancreatic beta cells for cytomegalovirus infection and the effects on cellular immunogenicity. Pancreas 41: 39–49. 22158077 10.1097/MPA.0b013e31821fc90c

[35] De Vos P, De Haan BJ, Van Schilfgaarde R (1997) Upscaling the production of microencapsulated pancreatic islets. Biomaterials 18: 1085–1090. 9247345 10.1016/s0142-9612(97)00040-9

[36] Vandenbossche GM, Van Oostveldt P, Remon JP (1991) A fluorescence method for the determination of the molecular weight cut-off of alginate-polylysine microcapsules. J Pharm Pharmacol 43: 275–277. 1712387 10.1111/j.2042-7158.1991.tb06683.x

[37] Vandenbossche GM, Van Oostveldt P, Demeester J, Remon JP (1993) The molecular weight cut-off of microcapsules is determined by the reaction between alginate and polylysine. Biotechnol Bioeng 42: 381–386. 18613023 10.1002/bit.260420316

[38] Coromili V, Chang TM (1993) Polydisperse dextran as a diffusing test solute to study the membrane permeability of alginate polylysine microcapsules. Biomater Artif Cells Immobilization Biotechnol 21: 427–444. 7691199 10.3109/10731199309117382

[39] De Haan BJ, van Goor H, De Vos P (2002) Processing of immunoisolated pancreatic islets: implications for histological analyses of hydrated tissue. Biotechniques 32: 612–614. 11911663 10.2144/02323rr03

[40] de Vos P, Smedema I, van Goor H, Moes H, van Zanten J, et al (2003) Association between macrophage activation and function of micro-encapsulated rat islets. Diabetologia 46: 666–673. 12750768 10.1007/s00125-003-1087-7

[41] Dijkstra CD, Dopp EA, Joling P, Kraal G (1985) The heterogeneity of mononuclear phagocytes in lymphoid organs: distinct macrophage subpopulations in the rat recognized by monoclonal antibodies ED1, ED2 and ED3. Immunology 54: 589–599. 3882559 PMC1453512

[42] Deenen GJ, Hunt SV, Opstelten D (1987) A stathmokinetic study of B lymphocytopoiesis in rat bone marrow: proliferation of cells containing cytoplasmic mu-chains, terminal deoxynucleotidyl transferase and carrying HIS24 antigen. JImmunol 139: 702–710. 3110280

[43] Huning T, Wallny HJ, Hartly J, Lawetsky A, Tiefenthaler G (1989) A monoclonal antibody to a constant region of the rat TCR that induces T-cell activation. JExpMed 169: 73–78. 10.1084/jem.169.1.73 PMC2189202 2783336

[44] de Vos P, Faas MM, Strand B, Calafiore R (2006) Alginate-based microcapsules for immunoisolation of pancreatic islets. Biomaterials 27: 5603–5617. 16879864 10.1016/j.biomaterials.2006.07.010

[45] De Vos P, De Haan B, Wolters GH, Van Schilfgaarde R (1996) Factors influencing the adequacy of microencapsulation of rat pancreatic islets. Transplantation 62: 888–893. 8878379 10.1097/00007890-199610150-00003

[46] de Vos P, Wolters GH, van Schilfgaarde R (1994) Possible relationship between fibrotic overgrowth of alginate-polylysine-alginate microencapsulated pancreatic islets and the microcapsule integrity. Transplant Proc 26: 782–783. 7513474

[47] de Vos P, Van Straaten JF, Nieuwenhuizen AG, de Groot M, Ploeg RJ, et al (1999) Why do microencapsulated islet grafts fail in the absence of fibrotic overgrowth? Diabetes 48: 1381–1388. 10389842 10.2337/diabetes.48.7.1381

[48] Schierbaum (1997) Hesse, M.: Meier, H.; Zech, B.: Spectroscopic Methods in Organic Chemistry (Translated by A. Linden, M. Murray). VIII and 365 pp., 221 fig., 100 tab., Hard cover: DM 168,–/SFr 149,–/ÖS 1226; ISBN 3 13 106 0611; Georg-Thieme Verlag Stuttgart – New York 1997; (New York ISBN 0 86577 6687). Starch - Stärke 49: 257–258.

[49] de Vos P, Hoogmoed CG, Busscher HJ (2002) Chemistry and biocompatibility of alginate-PLL capsules for immunoprotection of mammalian cells. J Biomed Mater Res 60: 252–259. 11857431 10.1002/jbm.10060

[50] Weir GC (2013) Islet encapsulation: advances and obstacles. Diabetologia 56: 1458–1461. 23636639 10.1007/s00125-013-2921-1

[51] Ratner BD, Bryant SJ (2004) Biomaterials: where we have been and where we are going. Annu Rev Biomed Eng 6: 41–75. 15255762 10.1146/annurev.bioeng.6.040803.140027

[52] Sawhney AS, Hubbell JA (1992) Poly(ethylene oxide)-graft-poly(L-lysine) copolymers to enhance the biocompatibility of poly(L-lysine)-alginate microcapsule membranes. Biomaterials 13: 863–870. 1457680 10.1016/0142-9612(92)90180-v

[53] Sawhney AS, Pathak CP, Hubbell JA (1993) Interfacial photopolymerization of poly(ethylene glycol)-based hydrogels upon alginate-poly(l-lysine) microcapsules for enhanced biocompatibility. Biomaterials 14: 1008–1016. 8286667 10.1016/0142-9612(93)90194-7

[54] Xu Y, Takai M, Ishihara K (2008) Suppression of Protein Adsorption on a Charged Phospholipid Polymer Interface. Biomacromolecules 10: 267–274. 10.1021/bm801279y 19090783

[55] Goto Y, Matsuno R, Konno T, Takai M, Ishihara K (2008) Polymer Nanoparticles Covered with Phosphorylcholine Groups and Immobilized with Antibody for High-Affinity Separation of Proteins. Biomacromolecules 9: 828–833. 18247529 10.1021/bm701161d

[56] Holland NB, Qiu Y, Ruegsegger M, Marchant RE (1998) Biomimetic engineering of non-adhesive glycocalyx-like surfaces using oligosaccharide surfactant polymers. Nature 392: 799–801. 9572137 10.1038/33894

[57] Bünger CM, Tiefenbach B, Jahnke A, Gerlach C, Freier T, et al (2005) Deletion of the tissue response against alginate-pll capsules by temporary release of co-encapsulated steroids. Biomaterials 26: 2353–2360. 15585238 10.1016/j.biomaterials.2004.07.017

[58] Tatarkiewicz K, Garcia M, Omer A, Van Schilfgaarde R, Weir GC, et al (2001) C-peptide responses after meal challenge in mice transplanted with microencapsulated rat islets. Diabetologia 44: 646–653. 11380084 10.1007/s001250051672

[59] Omer A, Keegan M, Czismadia E, de Vos P, Van Rooijen N, et al (2003) Macrophage depletion improves survival of porcine neonatal pancreatic cell clusters contained in alginate macrocapsules transplanted into rats. Xenotransplantation 10: 240–251. 12694544 10.1034/j.1399-3089.2003.01150.x

[60] de Vos P, van Hoogmoed CG, de Haan BJ, Busscher HJ (2002) Tissue responses against immunoisolating alginate-PLL capsules in the immediate posttransplant period. J Biomed Mater Res 62: 430–437. 12209929 10.1002/jbm.10345

[61] De Vos P, De Haan B, Pater J, Van Schilfgaarde R (1996) Association between capsule diameter, adequacy of encapsulation, and survival of microencapsulated rat islet allografts. Transplantation 62: 893–899. 8878380 10.1097/00007890-199610150-00004

[62] van Schilfgaarde R, de Vos P (1999) Factors influencing the properties and performance of microcapsules for immunoprotection of pancreatic islets. J Mol Med 77: 199–205. 9930963 10.1007/s001090050336

[63] Rokstad AM, Brekke O-L, Steinkjer B, Ryan L, Kolláriková G, et al (2011) Alginate microbeads are complement compatible, in contrast to polycation containing microcapsules, as revealed in a human whole blood model. Acta Biomater 7: 2566–2578. 21402181 10.1016/j.actbio.2011.03.011

[64] Orive G, Tam SK, Pedraz JL, Hallé J-P (2006) Biocompatibility of alginate–poly-l-lysine microcapsules for cell therapy. Biomaterials 27: 3691–3700. 16574222 10.1016/j.biomaterials.2006.02.048

[65] Tam SK, Bilodeau S, Dusseault J, Langlois G, Halle JP, et al (2011) Biocompatibility and physicochemical characteristics of alginate-polycation microcapsules. Acta Biomater 7: 1683–1692. 21145438 10.1016/j.actbio.2010.12.006

[66] King GA, Daugulis AJ, Faulkner P, Goosen MFA (1987) Alginate-Polylysine Microcapsules of Controlled Membrane Molecular Weight Cutoff for Mammalian Cell Culture Engineering. Biotechnology Progress 3: 231–240.

[67] Vandenbossche GMR, Bracke ME, Cuvelier CA, Bortier HE, Mareel MM, et al (1993) Host Reaction against Alginate-polylysine Microcapsules Containing Living Cells. J Pharm Pharmacol 45: 121–125. 8095526 10.1111/j.2042-7158.1993.tb03695.x

[68] Sobol M, Bartkowiak A, de Haan B, de Vos P (2013) Cytotoxicity study of novel water-soluble chitosan derivatives applied as membrane material of alginate microcapsules. J Biomed Mater Res A 101: 1907–1914. 23203606 10.1002/jbm.a.34500

[69] Skjåk-Bræk G, Murano E, Paoletti S (1989) Alginate as immobilization material. II: Determination of polyphenol contaminants by fluorescence spectroscopy, and evaluation of methods for their removal. Biotechnol Bioeng 33: 90–94. 18587847 10.1002/bit.260330112

[70] Paredes-Juarez GA, de Haan BJ, Faas MM, de Vos P (2013) The role of pathogen-associated molecular patterns in inflammatory responses against alginate based microcapsules. J Control Release 172: 983–992. 24051034 10.1016/j.jconrel.2013.09.009

[71] Thu B, Bruheim P, Espevik T, Smidsrod O, Soon-Shiong P, et al (1996) Alginate polycation microcapsules. I. Interaction between alginate and polycation. Biomaterials 17: 1031–1040. 8736740 10.1016/0142-9612(96)84680-1

[72] Stokke BT, Smidsroed O, Bruheim P, Skjaak-Braek G (1991) Distribution of uronate residues in alginate chains in relation to alginate gelling properties. Macromolecules 24: 4637–4645.

[73] Thu B, Bruheim P, Espevik T, Smidsrød O, Soon-Shiong P, et al (1996) Alginate polycation microcapsules: II. Some functional properties. Biomaterials 17: 1069–1079. 8718966 10.1016/0142-9612(96)85907-2

[74] Thu B, Skjåk-Bræk G, Micali F, Vittur F, Rizzo R (1997) The spatial distribution of calcium in alginate gel beads analysed by synchrotron-radiation induced X-ray emission (SRIXE). Carbohydr Res 297: 101–105.

